# Tailored Time–Temperature Transformation Diagram for IN718 Alloy Obtained via Powder Bed Fusion Additive Manufacturing: Phase Behavior and Precipitation Dynamic

**DOI:** 10.3390/ma16237280

**Published:** 2023-11-22

**Authors:** Julio Cesar Franco-Correa, Enrique Martínez-Franco, Celso Eduardo Cruz-González, Juan Manuel Salgado-López, Jhon Alexander Villada-Villalobos

**Affiliations:** 1 Posgrado Interinstitucional en Ciencia y Tecnología PICYT, CIDESI, Queretaro 76125, Mexico; jczfrancoc@gmail.com; 2 Center for Engineering and Industrial Development, CIDESI, Av. Pie de la Cuesta 702, Santiago de Querétaro 76125, Mexico; enrique.martinez@cidesi.edu.mx (E.M.-F.); ecruz@cidesi.edu.mx (C.E.C.-G.); msalgado@cidesi.edu.mx (J.M.S.-L.); 3 Consejo Nacional de Humanidades, Ciencia y Tecnología (CONAHCYT), Av. Insurgentes Sur 1582, Col. Crédito Constructor, Demarcación Territorial Benito Juárez, Mexico City 03940, Mexico

**Keywords:** IN718, stable and metastable phases, powder bed fusion (PBF), thermodynamic behavior, additive manufacturing (AM), phase transformations

## Abstract

Experimental and computational approaches were used to study the microstructure of IN718 produced via powder bed fusion additive manufacturing (PBF-AM). The presence, chemical composition, and distribution of stable and metastable phases (γ′′, δ, MC, and Laves) were also analyzed. The information obtained from the microstructural study was used to construct a tailored time–temperature transformation (TTT) diagram customized for additive manufacturing of IN718. Experimental techniques, including differential scanning calorimetry (DSC), scanning electron microscopy, energy dispersive X-ray spectroscopy, and electron backscatter diffraction (EBSD), were employed to establish the morphological, chemical, and structural characteristics of the microstructure. The Thermo-Calc software and a Scheil–Gulliver model were used to analyze the presence and behavior of phase transformations during heating and cooling processes under non-thermodynamic equilibrium conditions, typical of AM processes. Unlike conventional TTT diagrams of this alloy, the diagram presented here reveals that the precipitation of γ′′ and δ phases occurs at lower temperatures and shorter times in AM-manufactured parts. Significantly, the superposition of γ′′ and δ phase curves in the proposed diagram underscores the interdependence between these phases. This TTT diagram is a valuable insight that can help in the development of heat treatment processes and quality control for IN718 produced via PBF-AM.

## 1. Introduction

Additive manufacturing (AM) introduces new production methods and allows customization of materials’ geometric shape, chemical composition, and microstructure, enhancing the performance of functionally graded materials (FGM) [1]. In recent years, researchers such as [2,3,4] have conducted experimental studies on phase transformations, melting and solidification structures, residual stress, and optimal processing parameters, revealing essential information on surface topography, grain structure, crystalline structure, phases, and precipitates. These studies have acquired correlated thermodynamics and kinetics behavior, diffusion phenomena, and thermal history in order to predict phase transformations in alloys obtained via AM. On the other hand, simulations in AM offer a powerful approach for extracting valuable information about material properties. Using thermodynamic modeling, such as CALPHAD methodology, it becomes feasible to predict equilibrium phases in a multi-component system [5].

IN718 is a nickel-based superalloy well known for its exceptional mechanical properties [6]. These exceptional properties are achieved via a precipitation hardening mechanism through the use of AMS5662 [7] and AMS5383 [8] heat treatments suitable for components manufactured using conventional methods such as forging or casting [9]. These treatments promote the precipitation of γ′ and γ′′ phases, which provides the material with exceptional properties of high resistance to stress, fatigue, rupture, and creep at high temperatures. However, in AM, the microstructural behavior of IN718 is influenced by several factors, including solidification under non-thermodynamic equilibrium conditions, high-temperature gradients, and thermal history during fabrication.

As a result, precipitation, solidification, and reinforcing phase growth mechanisms can exhibit notable differences compared to traditional manufacturing methods. In their study, Sanchez et al. [10] show a comprehensive summary of the factors impacting the functional performance of nickel-based superalloys specifically manufactured using additive processes. Morphological defects and microstructures are present and differ from those obtained using conventional manufacturing techniques. Moreover, microstructural characteristics and mechanisms such as phase precipitation, textures, and grain sizes vary from reported thermodynamic predictions. Gallmeyer et al. [11] report that, during the formation of parts obtained via AM, the precipitation of the δ phase is detrimental and undesirable during the nucleation process of reinforcing phases such as γ’ and γ′′. Naiyuan Xi et al. [12] established that the configurations of the δ phase have a significant effect on the plastic deformation of grains, where the large δ phase at the grain boundary limits the intergranular deformation significantly, but the large intragranular δ phase may cause strain localization and then lead to rapid failure. In summary, the kind, morphology, and location of the phases in the AM process make it difficult to understand the solidification process of the reinforcing phases and, consequently, its impact on mechanical properties.

In a previous work published by our team [13], it has been demonstrated that the effect of conventional heat treatment [7,8] is different when it is applied to AM-produced parts of IN718. For this reason, we decided to go further in the investigation, looking for a TTT diagram that represents the phase transformation as a function of the time and temperature for AM-produced IN718.

In this paper, experimental and computational approaches were used to study the microstructure of IN718 produced via powder bed fusion additive manufacturing (PBF-AM). The presence, chemical composition, and distribution of stable and metastable phases (γ′′, δ, MC, and Laves) were also analyzed. The information obtained from the microstructural study (grain size and the composition, location, and distribution of the phases) was used to construct a tailored time–temperature transformation (TTT) diagram customized for additive manufacturing of IN718. Unlike conventional TTT diagrams for this alloy, the diagram presented here reveals that the precipitation of the γ′′ and δ phases occurs at lower temperatures and for shorter times in AM-manufactured parts. Significantly, the superposition of the γ′′ and δ phase curves in the proposed diagram underscores the interdependence between these phases. The results could be used as a reference for applying a heat treatment to an AM-produced IN718 alloy.

## 2. Materials and Methods

**Figure 1:**
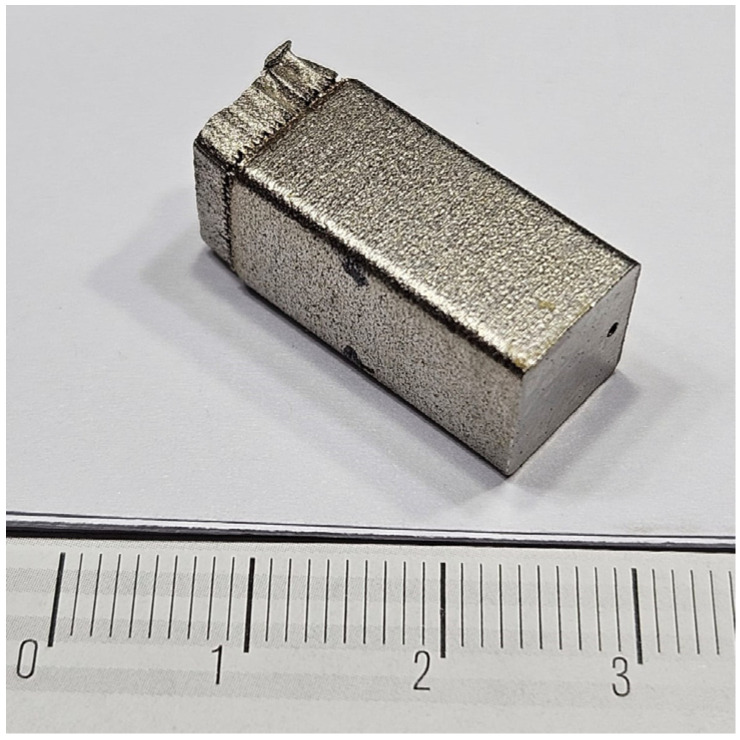
Sample of IN718 manufactured via PBF.

Figure 1 shows a typical IN718 sample manufactured using powder bed fusion (PBF) technology with an EOSINT M280 3D printer (manufactured by EOS GmbH, Krailling, Germany) in an inert atmosphere (Argon). The printing parameters were optimized to produce samples 10 mm × 10 mm × 10 mm in size with a layer thickness of 0.040 mm, a hatch distance of 0.110 mm, a scan speed of 960 mm/s, a laser power of 285 W, and a 67° layer rotation. The chemical composition of the powder was analyzed using a Varian SpectrAA 220 Atomic Absorption Spectrometer (from Varian, Alto Palo, CA, USA). The measured composition of the powder was as follows: Ni (50.28%), Fe (Balance), Cr (18.27%), Nb (7.27%), Mo (3.48%), Ti (1.1%), Al (0.45%), and C (0.08%). Thermo-Calc^®^ software (https://thermocalc.com (accessed on 13 November 2023), Stockholm, Sweden) version 2019.1, which uses the CALPHAD method, was utilized to predict the phase equilibria in multi-component systems. The Thermo-Calc Nickel-based superalloys databases (TCNI8, MOBNI4) were used to calculate phase fractions, Scheil–Gulliver solidification, and diagrams in temperatures ranging from 300 to 1400 °C. The simulation parameters for AM-manufactured goods, such as dislocation density alloys or the wetting angle, were adopted from the literature [14,15]. Experimental data on morphology and microstructure used in the simulations were obtained from our previous investigation on IN718 [13].

The metallographic preparation of the as-built bulk material was carried out. First, 50 mg of material was obtained using a cutting disk from Buehler (Lake Bluff, IL, USA). Then, this sample was subjected to thermal analysis using a SETSYS Evolution DTA/DSC instrument (Setaram Instrumentation, Caluire-et-Cuire, Francia) according to ASTM D3418 [16] to measure thermal behavior from 25 °C to 1400 °C. The sample was heated at a rate of 10 °C/min and was soaked for 5 min at 1400 °C to homogenize it; then, it was cooled at the same rate.

After the thermal analysis, the sample was prepared for metallographic characterization. This process involved manual grinding using SiC abrasive paper with grit sizes ranging from 320 to 2000, followed by polishing down to 0.5 μm using an alcohol-based diamond suspension and a VibroMet™ machine (Buehler, IL, USA) for 24 h. A Kallings 2 etching step was then applied according to ASTM E407-07 [17], and any residual scratches and deformed traces were eliminated using an IM4000Plus HITACHI ion milling system (Hitachi, Tokyo, Japan) with an accelerating voltage of 6 kV and a rotation speed of 25 rpm for 40 min.

An SEM SU-8230 HITACHI (Hitachi, Tokyo, Japan) equipped with a Bruker e-Flash HR+ electron backscatter diffraction (EBSD) detector was used to analyze the microstructure, texture, grain size, and distribution at 15 Kv and magnification between ×35.0 K and ×150 K. Using EBSD analysis, high-resolution images were acquired at a magnification of ×700, a step size of 1.0 um, and a resolution of 512 × 416 pixels. A grain confidence index standardization was implemented, with angle tolerance set at 10° to ensure accurate and reliable results. Moreover, a neighbor confidence index correlation was considered, requiring a threshold of ≥0.1 and a neighbor orientation correlation.

## 3. Results and Discussion

### 3.1. Phase Dynamics versus Temperature

**Figure 2:**
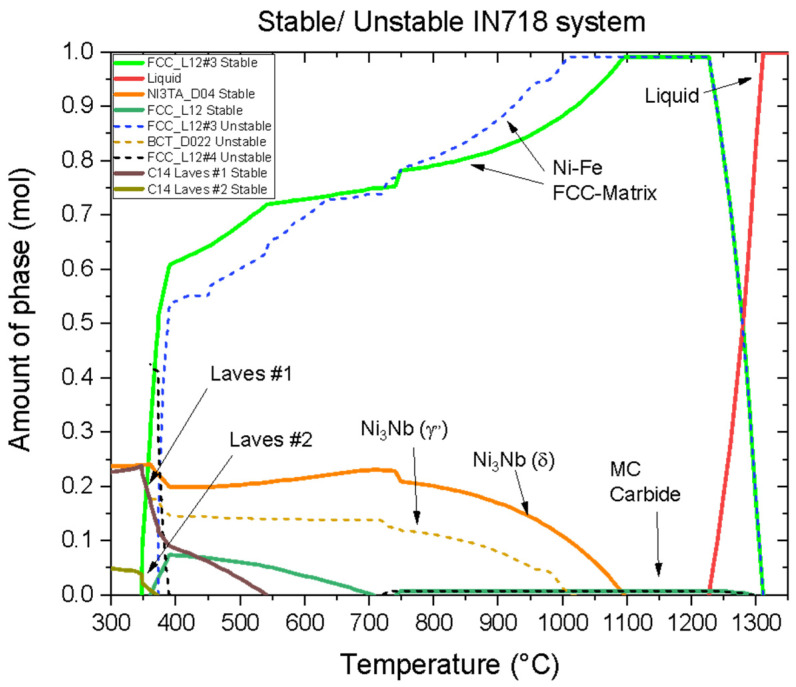
Prediction of phase amounts in the IN718 system for the investigated chemical composition. The graph showcases stable phases (solid lines) and metastable phases (dashed lines).

Figure 2 illustrates the prevailing phases and precipitates observed in the IN718 alloy, obtained from Thermo-Calc^®^ software (https://thermocalc.com (accessed on 13 November 2023)) (Stockholm, Sweden). At temperatures below 400 °C, the presence of Laves phases becomes evident. The formation of these intermetallic phases results from the kinetic process of the PBF manufacturing process, thermal history, and the availability of chemical elements. At temperatures above 400 °C, the alloy can exhibit the coexistence of stable (δ) and metastable (γ′′) phases with Ni3Nb stoichiometry. The prevalence of each phase is strongly influenced by the availability of chemical elements and the temperature gradient experienced during the manufacturing process. Temperatures exceeding 725 °C reveal the precipitation of MC carbides, mainly NbC. The growth of this carbide is dependent upon the availability of Nb present in the δ and γ′′ phases. At the same time, the presence of available Ni facilitates an increase of the Ni-Fe matrix phase. The impact of these dissolutions becomes evident through observable slope changes in the quantities of the matrix phase (green line), the δ phase (orange line), and the γ′′ phase (dashed orange line).

The presence of stable and metastable phases in IN718 plays a crucial role in determining its mechanical properties. The metastable γ′′ phase, in particular, acts as the primary reinforcing precipitate, significantly enhancing the mechanical properties of the alloy. However, over time and with exposure to elevated temperatures during service conditions, this metastable phase transforms into the stable phase δ. This phase transformation leads to changes in the mechanical properties of the material, often resulting in a decrease in strength and potentially affecting other properties.

According to Rayner et al. [18], there is a relationship between DSC temperature peaks and phase transformations. In AM, the thermal characteristics of phase diagrams where there are non-equilibrium thermodynamic conditions cause the formation of metastable phases. Thus, the Scheil–Gulliver solidification model provides an accurate transition temperature of the material, determining heat signals associated with the formation of phases [19].

**Figure 3:**
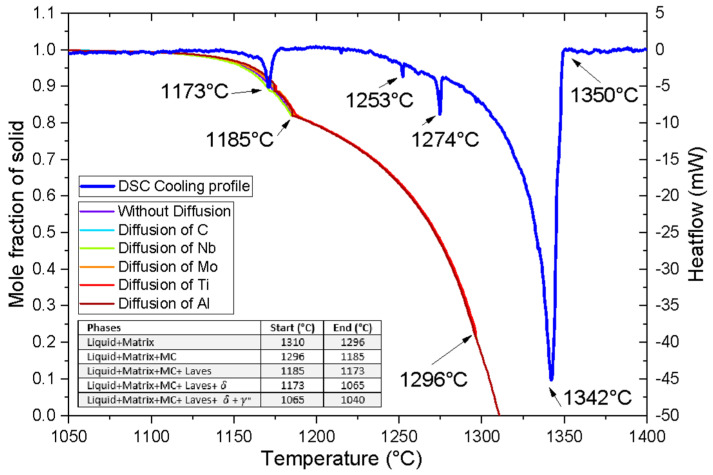
Solidification path under Scheil–Gulliver model (with and without diffusion of alloys) and experimental DSC cooling profile (thick blue line) of IN718 obtained via AM.

In Figure 3, the simulation of the solidification profile of the IN718 alloy reveals that the formation of MC carbides starts at a temperature below 1296 °C. In addition, the simulation also supports the preference for the precipitation of NbC, which is in good agreement with experimental findings, exhibiting peaks at 1274 and 1253 °C, as reported by other authors [20,21], where all identifications were based on the solidification pattern Liquid → Liquid + γ → Liquid + γ + NbC → Liquid + γ + NbC + Laves.

Within the temperature range of 1185 to 1173 °C, the formation of Laves phases is distinctly detected. As the temperature falls below 1173 °C, the availability of chemical elements facilitates the stoichiometric formation of the Ni_3_Nb phase. However, precisely determining the proportions of γ″ and δ phases in this region remains challenging despite the Scheil–Gulliver model’s prediction of the formation of γ′′. Particularly, simulations involving diffusive elements show minimal modification to the transformations around 1173 °C. The Scheil–Gulliver model is a widely utilized approach for simulating the solidification behavior of alloys [22]. It is well known that non-equilibrium solidification can induce changes to the solidus temperature due to microsegregation. This phenomenon manifests as variations in composition at the arms of dendrites, and the final part of the liquid solidifies in interdendritic regions with high concentrations of some alloying elements. The model accurately predicts the temperature of phase transformations, but it is important to consider that microsegregation can lead to variations in precipitation temperatures when different diffusive elements are considered. For example, in IN718, low-solubility elements such as Nb, Mo, Ti, and C tend to segregate at the interdendritic regions and form MC carbides and Laves phases [23]. These discrepancies have been observed and reported in several studies, including the research conducted by Knorovsky et al. [24].

The solidification process is a complex phenomenon subject to the influence of various factors, such as the kinetics of solute diffusion, the thermodynamics of microsegregation, and cooling conditions. In the simulation, the diffusion of a single element (Nb, Mo, Al, Ti, and C) was considered, while the other elements followed the classic behavior of the Scheil–Gulliver model. Consequently, it became essential to account for additional factors, including the multicomponent system’s kinetics and the manufacturing technique’s impact on dendrite geometry and thermal history, to comprehensively understand the process.

**Figure 4:**
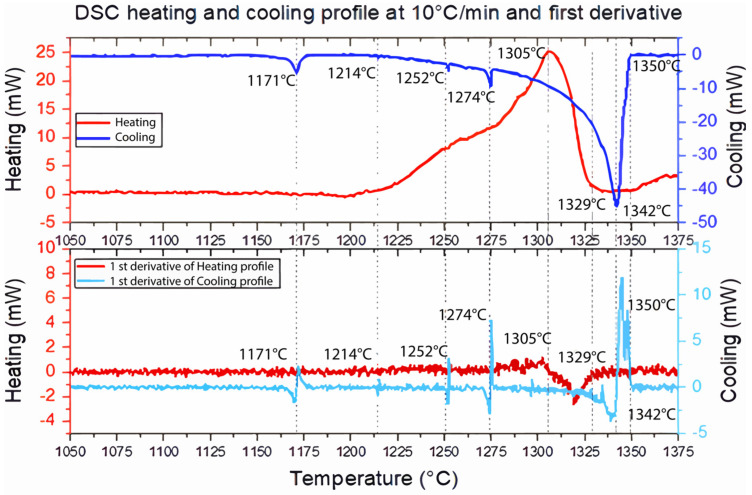
DSC heating and cooling curve profiles at 10 °C/min and the first derivative for IN718 obtained via AM. Several peaks are observed during the cooling process, and a broad peak is observed during the heating. Peak temperatures represent a phase transformation.

Identifying endothermic and exothermic peaks during DSC analysis is crucial in determining critical temperatures, such as the liquidus, eutectic, and nucleation temperatures. In Figure 4, a broader peak emerges during the heating profile near 1305 °C. The onsets of multiple peaks overlap due to solid solution partial melting, as reported by Han et al. [15], resulting in a broad peak with changes in its slope. On the other hand, during the cooling profile near 1342 °C, a broad peak is accompanied by three lower-height peaks: the peaks at 1252 °C and 1274 °C correspond to MC carbides, while the peak at 1171 °C corresponds to the Laves phase, following the path reported by Shi et al. [20]. Heating and cooling enthalpy values of 4.698 mJ/K × mol and 4.596 mJ/K × mol, respectively, were obtained by measuring the area under the curve. This difference is attributed to the exothermic contribution of the three peaks in the total solidification energy associated with the Laves phase and the precipitation of γ′′, δ, and MC phases during cooling [25].

### 3.2. Microstructural Characterization

The manufacturing process conditions deeply influence the microstructure’s morphology and crystalline characteristics. As such, it becomes crucial to accurately characterize the microstructural settings to gain a comprehensive understanding of the mechanical properties exhibited by AM-produced samples. Scanning electron microscopy (SEM) and EBSD were employed to identify both morphology and crystallographic orientations within the microstructure.

**Figure 5:**
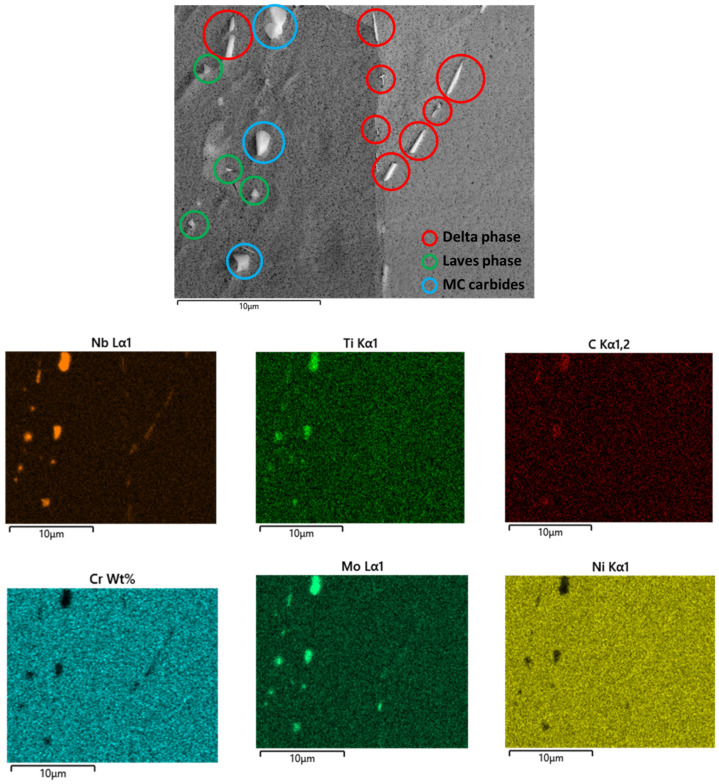
SEM and EDS analysis of the microstructure of IN718. The MC precipitates and Laves, and δ phases were identified through chemical composition, morphology, and distribution.

Figure 5 offers valuable insights into the microstructure, revealing the presence of MC precipitates and Laves phases positioned intragranularly, distinguished by diameters ranging from 0.5 to 2 µm, as reported to occur by Zhao et al. [26] when the high cooling rate during rapid solidification results in the simultaneous formation of Laves phases and MC carbides. Nb and Mo promote Laves formation, while Nb and C promote the precipitation of NbC in interdendritic zones.

The presence of Laves phases in the IN718 alloy significantly impacts its behavior and composition during additive manufacturing, particularly in processes like powder bed fusion. Laves phases are Nb-rich intermetallic compounds. The key consequence of the high concentration of Laves phases is the depletion of Nb in the solid solution, which plays a critical role in the precipitation of the γ′′ phase. Since there is insufficient Nb available for γ′′ formation, the primary reinforcing phase in this alloy, mechanical properties are notably affected. This depletion of Nb and the consequent alteration in the alloy’s microstructure can result in reduced strength, toughness, and other mechanical characteristics.

Carbides are located in different zones of the microstructure, within the grain, and at the grain boundaries. The first ones reduce dislocation movement inside the grains, improving creep resistance and hardness. On the other hand, carbides at the grain boundaries could be of two types: primary (MC) or secondary carbides (M_23_C_6_). The primary carbides reduce grain sliding, improving the creep resistance. Meanwhile, the secondary carbides reduce the mechanical properties because they promote intergranular cracking. The control of the type and location of carbides can be applied to control the final mechanical properties after heat treatment.

High cooling rates reduce Nb segregation and the extent of Laves formation. A distinct δ phase is observed at the grain boundaries, characterized by a needle-like morphology with lengths of approximately 1 µm. These findings align consistently with the δ phase prominently detected by DSC measures and the Scheil–Gulliver model simulation at temperatures below 1173 °C.

**Figure 6:**
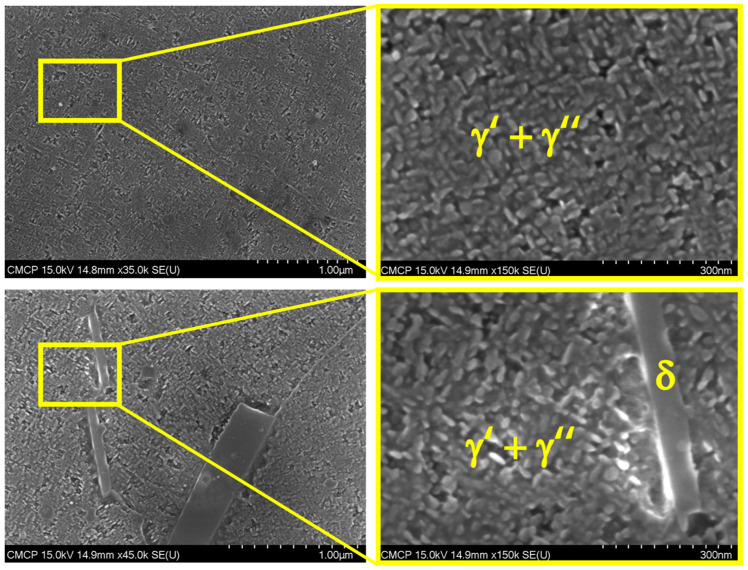
High-magnification SEM images provide a detailed view of the microstructure of IN718. Notably, reinforcing phases are discerned at the nanometer scale, contrasting with the larger-scale δ phase.

Figure 6 reveals the presence of γ′′, γ′, and δ phases despite these not being detected by DSC measures. Laves phases constitute approximately 1 to 2% of the microstructural composition, while NbC carbides contribute 8%. The presence of reinforcing phases γ′′, MC carbides, and Laves phases was confirmed using EBSD, SEM, and EDS in interdendritic positions, while the δ phase was found to be located at grain boundaries. This information is consistent with that reported by Hasani et al. [27], who state that the fact δ phase exhibits preferential nucleation at grain boundaries can be attributed to the higher concentration of Nb in these regions. Additionally, δ phase growth predominantly occurs along the grain boundaries, manifesting as needle-like structures, and is observed in a limited number of grain interiors at lower temperatures.

**Figure 7:**
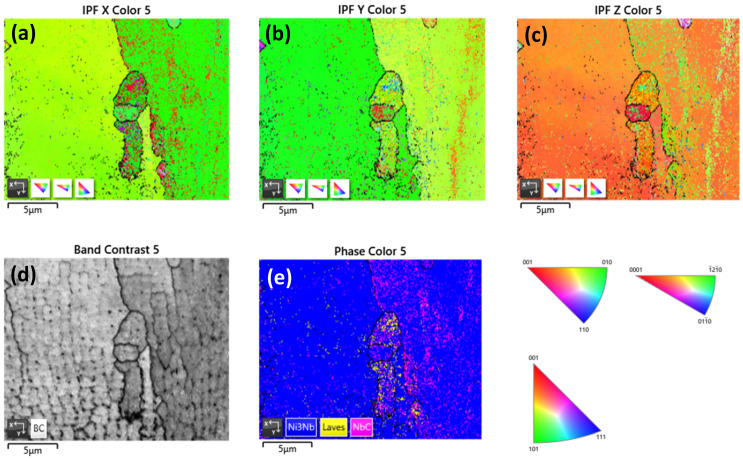
EBSD inverse pole figure (IPF) in three axes (**a**) x, (**b**) y, (**c**) z. (**d**) SEM image, and (**e**) phase distribution of γ′′, Laves, and NbC carbides obtained at 20 Kv and tilt 70°.

EBSD analysis (Figure 7) was conducted to identify Ni_3_Nb, Laves, and NbC carbides within the microstructure. The γ′′, γ′, and the matrix were collectively referred to as Ni_3_Nb due to their similar FCC structure and lattice parameters. The coherence between these phases limits the EBSD technique’s ability to determine their distribution and crystal orientation accurately [28]. A comparison with SEM morphological shapes from previous studies was carried out to confirm the presence of Laves and NbC phases [14]. For identification, the lattice parameters in Å were used: Ni_3_Nb (a = 3.62, b = 3.62, c = 7.41), Laves (a = 5.01, b = 5.01, c = 8.06), and NbC (a = 4.40, b = 4.40, c = 4.40). The IPF images revealed a strong orientation of Ni_3_Nb on the (010) plane within the grains. Moreover, the grain boundaries exhibited a higher concentration of NbC and Laves phases with distinct orientations. SEM imaging facilitated the observation of grain boundaries and anisotropic grains. The phase distribution analysis showed that NbC carbides were more prevalent within the grains compared to Laves phases.

The presence of the δ phase at the grain boundary negatively impacted the deformation capacity, resulting in stress/strain concentration around the grain boundary region, as discussed by Rielli et al. [23].

### 3.3. TTT Diagram

Several studies, including [29,30], have extensively investigated the TTT (time–temperature transformation) phase diagrams of IN718 to understand the precipitation kinetics of δ and γ′′ phases. A TTT diagram determines the dominance of either the driving force or atomic mobility in the formation of phases and their volume fraction [31].

Accurately predicting the phase precipitation is essential for controlling microstructure and mechanical properties, regardless of the manufacturing process. However, the segregation of Nb is significantly influenced by the diffusion of alloys in both interdendritic and dendritic regions, as reported here and by other authors [32,33]. Therefore, it is imperative to consider the thermal history, the initial microstructure, and the chemical composition to construct a reliable TTT diagram that accurately predicts the precipitation of phases.

**Figure 8:**
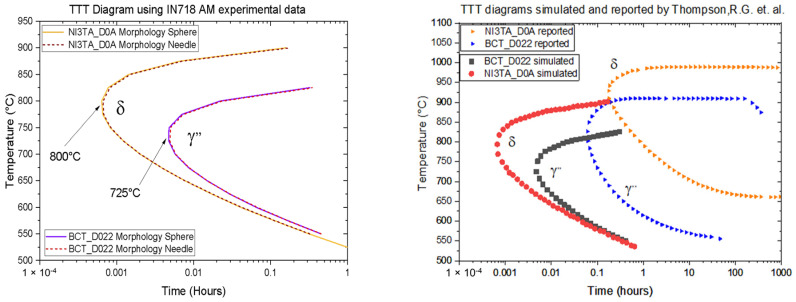
(**Left**) δ and γ′′ TTT diagram of IN718 obtained via AM according to morphology shape (sphere and needle) when nucleating in bulk. (**Right**) Comparison between our simulated TTT diagram of IN718 and others referenced [34].

Figure 8 compares the precipitation of the stable δ phase and the metastable γ′′ phase based on temperature and exposure time. The curves show no apparent difference in the shape of the “nose” between the spherical (continuous line) and needle-like (dotted line) morphologies. Moreover, it provides a comparison between the TTT diagram obtained in this work for AM and the TTT diagram for conventional manufacturing published by Thompson et al. [34]. The difference between the two TTT diagrams results mainly from the varying chemical compositions and the methods of manufacture. These factors influence the thermal gradient and dendritic growth in the microstructure, thus modifying the position of the nose and shape of the profile by shifting it to the left of the diagram, which indicates faster transformations due to the rapid cooling that occurs during solidification in additive manufacturing.

The generation of TTT diagrams is affected by various solidification conditions, such as the cooling rate, the dendritic structure, the precipitation kinetics in dendritic and inter-dendritic zones, the availability, and local variations of chemical elements for precipitation, which play significant roles in shaping and positioning the nose of the TTT diagram [32,33]. The TTT phase diagram proposed in this study is a valuable reference tool for comprehending and predicting the solidification process, offering critical insights for controlling and enhancing microstructural properties in additive-manufactured IN718. By accounting for a range of thermal and temporal parameters, this diagram facilitates informed decision-making throughout the manufacturing process, ensuring precise control over desired phase transformations and ultimately enhancing the overall performance of IN718 components produced via additive manufacturing.

## 4. Conclusions

In this study, we conducted both experimental investigations and simulations to gain deeper insights into the thermodynamic and microstructural behavior of the IN718 alloy produced via additive manufacturing. Our findings have led to several conclusions:

1. The study successfully predicted the presence of stable and metastable phases and precipitates for a specific chemical composition of the alloy. However, the complex PBF manufacturing process induced intricate phase formations, including Laves phases, which competed with stable and metastable phases such as δ and γ′′, ultimately influencing the chemical composition of the matrix.
2. Using DSC analysis and correlation with the Scheil–Gulliver solidification model, we highlighted the predominant formation of MC carbides, a result that was further supported by experimental findings. The presence of Laves phases had significant implications for the availability of chemical elements. Although quantifying the exact proportions of γ′′ and δ phases in a specific range proved challenging, the correlations provided valuable insights.
3. Using SEM/EDX/EBSD, it was possible to successfully morphologically identify phases and precipitates based on their distinctive shapes, chemical compositions, and spatial distribution within the microstructure. The complexity of the AM production process significantly influenced the microstructure’s morphological and crystalline features, emphasizing the need for a thorough understanding of these conditions to assess the mechanical properties accurately. The confirmation of Laves, MC carbides, and δ phases through various analytical techniques underscored the complexity of the microstructure.
4. Furthermore, we showed that a TTT diagram accurately predicts the solidification process of the δ and γ′′ for IN718 superalloy under AM conditions. The comparison between the precipitation of the stable δ phase and the metastable γ′′ highlighted the limitations of relying solely on TTT diagrams to depict the complete kinetic behavior of AM-produced samples. Local variations in chemical composition and other factors influenced thermal gradients and dendritic growth in the microstructure, calling for further research to comprehensively understand additive manufacturing and accurately evaluate its mechanical properties.
5. The tailored TTT diagram provides critical information about the phase transformations and microstructural evolution that occur during the heat treatment of components produced by additive manufacturing. With this knowledge, engineers can design heat treatment cycles specifically suited for the AM-produced components. By adjusting the temperature, holding times, and cooling rates based on the TTT diagram, they can optimize the microstructure to achieve the desired material properties, such as strength, hardness, and ductility.

Our comprehensive approach, combining experiments and simulations, has significantly advanced the understanding of the IN718 alloy’s behavior in additive manufacturing settings. The knowledge gained from this study can contribute to controlling the microstructural properties of AM materials for various engineering applications.

## Data Availability

Data is contained within the article.

## References

[1] Tan C., Weng F., Sui S., Chew Y., Bi G. (2021). Progress and perspectives in laser additive manufacturing of critical aeroengine materials. Int. J. Mach. Tools Manuf..

[2] Ghanavati R., Naffakh-Moosavy H. (2021). Additive manufacturing of functionally graded metallic materials: A review of experimental and numerical studies. J. Mater. Res. Technol..

[3] Chen S.G., Gao H.J., Wu Q., Gao Z.H., Zhou X. (2022). Review on residual stresses in metal additive manufacturing: Formation mechanisms, parameter dependencies, prediction and control approaches. J. Mater. Res. Technol..

[4] Cooke S., Ahmadi K., Willerth S., Herring R. (2020). Metal additive manufacturing: Technology, metallurgy, and modeling. J. Manuf. Process..

[5] Lindwall G., Campbell C.E., Lass E.A., Zhang F., Stoudt M.R., Allen A.J., Levine L.E. (2019). Simulation of TTT curves for additively manufactured Inconel 625. Metall. Mater. Trans. A Phys..

[6] Paulonis D.F., Schirra J.J. (2001). Alloy 718 at Pratt & Whitney-Historical Perspective and Future Challenges. Superalloys 718, 625, 706 and Various Derivatives.

[7] (2016). Nickel Alloy, Corrosion and Heat-Resistant, Bars, Forgings, and Rings 52.5Ni-19Cr-3.0Mo-5.1Cb (Nb)-0.90Ti-0.50AI-18Fe Consumable Electrode or Vacuum Induction Melted 1775 °F (968 °C) Solution Heat Treated, Precipitation Hardenable—UNS N07718.

[8] (2018). Nickel Alloy, Corrosion and Heat-Resistant, Investment Castings 52.5Ni-19Cr-3.0Mo-5.1Cb(Nb)-0.90Ti-0.60Al-18Fe Vacuum Melted Homogenization and Solution Heat Treated.

[9] Trosch T., Strößner J., Völkl R., Glatzel U. (2016). Microstructure and Mechanical Properties of Selective Laser Melted Inconel 718 Compared to Forging and Casting. Mater. Lett..

[10] Sanchez S., Smith P., Xu Z., Gaspard G., Hyde C.J., Wits W.W., Ashcroft I.A., Chen H., Clare A.T. (2021). Powder Bed Fusion of nickel-based superalloys: A review. Int. J. Mach. Tools Manuf..

[11] Gallmeyer T.G., Moorthy S., Kappes B.B., Mills M.J., Amin-Ahmadi B., Stebner A.P. (2020). Knowledge of process-structure-property relationships to engineer better heat treatments for laser powder bed fusion additive manufactured Inconel 718. Addit. Manuf..

[12] Xi N., Ni Z., Fang X., Zhou Y., Tang K., Zhang H., Huang K. (2023). Role of δ-phase on Mechanical Behaviors of Additive Manufactured Inconel 718: Detailed Microstructure Analysis and Crystal Plasticity Modelling. Int. J. Plast..

[13] Franco-Correa J.C., Martínez-Franco E., Alvarado-Orozco J.M., Cáceres-Díaz L.A., Espinosa-Arbelaez D.G., Villada J.A. (2021). Effect of Conventional Heat Treatments on the Microstructure and Microhardness of IN718 Obtained by Wrought and Additive Manufacturing. J. Mater. Eng. Perform..

[14] De Jaeger J., Solas D., Baudin T., Fandeur O., Schmitt J.H., Rey C. (2012). Inconel 718 single and multipass modelling of hot forging. Proceedings of the Superalloys 2012: 12th International Symposium on Superalloys.

[15] Han W., Wan M., Zhao R., Kang H., Rao Y. (2022). Microstructural evolution and mechanical properties of brazed IN718 ultrathin-walled capillary structure using different particulate reinforced filler alloy. Chin. J. Aero..

[16] (2021). 12 Standard Test Method for Transition Temperatures and Enthalpies of Fusion and Crystallization of Polymers by Differential Scanning Calorimetry.

[17] (2015). Standard Practice for Microetching Metals and Alloys.

[18] Rayner A.J., Corbin S.F. (2021). Grain growth activation during supersolidus liquid phase sintering in a metal injection molded nickel-base superalloy. Mater. Today Commun..

[19] Shafiee A., Moon J., Kim H.S., Jahazi M., Nili-Ahmadabadi M. (2019). Precipitation behaviour and mechanical properties of a new wrought high entropy superalloy. Mater. Sci. Eng. A.

[20] Shi X., Duan S., Yang W., Guo H., Guo J. (2018). Solidification and segregation behaviors of superalloy IN718 at a slow cooling rate. Materials.

[21] Miao Z.J., Shan A.D., Wu Y.B., Jun L.U., Ying H.U., Liu J.L., Song H.W. (2012). Effects of P and B addition on as-cast microstructure and homogenization parameter of Inconel 718 alloy. Trans. Nonferrous Met. Soc. China.

[22] Vyazovkin S., Koga N., Schick C. (2018). Handbook of Thermal Analysis and Calorimetry: Recent Advances, Techniques, and Applications.

[23] Rielli V.V., Piglione A., Pham M.S., Primig S. (2022). On the detailed morphological and chemical evolution of phases during laser powder bed fusion and common post-processing heat treatments of IN718. Addit. Manuf..

[24] Knorovsky G.A., Cieslak M.J., Headley T.J., Romig A.D., Hammetter W.F. (1989). Inconel 718: A solidification diagram. Metall. Mater. Trans. A Phys..

[25] Tang Y.T., Panwisawas C., Ghoussoub J.N., Gong Y., Clark J.W., Németh A.A., McCartney D.G., Reed R.C. (2021). Alloys-by-design: Application to new superalloys for additive manufacturing. Acta Mater..

[26] Zhao Z., Li L., Yang W., Zeng Y., Lian Y., Yue Z. (2022). A comprehensive study of the anisotropic tensile properties of laser additive manufactured Ni-based superalloy after heat treatment. Int. J. Plast..

[27] Hasani N., Dharmendra C., Sanjari M., Fazeli F., Amirkhiz B.S., Pirgazi H., Janaki-Ram G.D., Mohammadi M. (2021). Laser powder bed fused Inconel 718 in stress-relieved and solution heat-treated conditions. Mater. Charact..

[28] Cao M., Zhang D., Gao Y., Chen R., Huang G., Feng Z., Poprawe R., Schleifenbaum J.H., Ziegler S. (2021). The effect of homogenization temperature on the microstructure and high temperature mechanical performance of SLM-fabricated IN718 alloy. Mater. Sci. Eng. A.

[29] Qin H., Bi Z., Yu H., Feng G., Zhang R., Guo X., Chi H., Du J., Zhang J. (2018). Assessment of the stress-oriented precipitation hardening designed by interior residual stress during aging in IN718 superalloy. Mater. Sci. Eng. A.

[30] Lin Y.Y., Schleifer F., Fleck M., Glatzel U. (2020). On the interaction between γ″ precipitates and dislocation microstructures in Nb containing single crystal nickel-base alloys. Mater. Charact..

[31] Zhang S., Wang L., Lin X., Yang H., Li M., Lei L., Huang W. (2021). Precipitation behavior of δ phase and its effect on stress rupture properties of selective laser-melted Inconel 718 superalloy. Composites Part B Eng..

[32] Jang J., Van D., Lee S.H. (2022). Precipitation kinetics of secondary phases induced by heat accumulation in the deposit of Inconel 718. Addit. Manuf..

[33] Yu X., Lin X., Tan H., Hu Y., Zhang S., Liu F., Yang H., Huang W. (2021). Microstructure and fatigue crack growth behavior of Inconel 718 superalloy manufactured by laser directed energy deposition. Int. J. Fatigue.

[34] Thompson R.G., Dobbs J., Mayo D. (1986). The effect of heat treatment on microfissuring in alloy 718. Weld J..

